# Sepsis Management in Southeast Asia: A Review and Clinical Experience

**DOI:** 10.3390/jcm11133635

**Published:** 2022-06-23

**Authors:** Yatin Mehta, Rajib Paul, Raihan Rabbani, Subhash Prasad Acharya, Ushira Kapilani Withanaarachchi

**Affiliations:** 1 Institute of Critical Care and Anesthesiology, Medanta the Medicity, Sector-38, Gurugram 22001, India; 2 Internal Medicine, Apollo Hospitals, Road Number 72, Jubilee Hills, Hyderabad 500033, India; drrajibpaul@gmail.com; 3 Critical Care & Internal Medicine, Square Hospitals Ltd., 18 Bir Uttam Qazi NuruzzamanSarak West, Panthapath, Dhaka 1205, Bangladesh; raihanrabbani@yahoo.com; 4 Critical Care Medicine, Institute of Medicine, Tribhuvan University, Maharajgunj, Kathmandu 44618, Nepal; drsuvash@gmail.com; 5 Paediatrics/NICU, Teaching Hospital Karapitiya, No. 124, Ananda Mawatha, Kithulampitiya, Galle 80000, Sri Lanka; kapilani@gmail.com

**Keywords:** sepsis, septic shock, COVID-19, colistin resistance and chloramphenicol boon, cytosorb

## Abstract

Sepsis is a life-threatening condition that causes a global health burden associated with high mortality and morbidity. Often life-threatening, sepsis can be caused by bacteria, viruses, parasites or fungi. Sepsis management primarily focuses on source control and early broad-spectrum antibiotics, plus organ function support. Comprehensive changes in the way we manage sepsis patients include early identification, infective focus identification and immediate treatment with antimicrobial therapy, appropriate supportive care and hemodynamic optimization. Despite all efforts of clinical and experimental research over thirty years, the capacity to positively influence the outcome of the disease remains limited. This can be due to limited studies available on sepsis in developing countries, especially in Southeast Asia. This review summarizes the progress made in the diagnosis and time associated with sepsis, colistin resistance and chloramphenicol boon, antibiotic abuse, resource constraints and association of sepsis with COVID-19 in Southeast Asia. A personalized approach and innovative therapeutic alternatives such as CytoSorb^®^ are highlighted as potential options for the treatment of patients with sepsis in Southeast Asia.

## 1. Introduction

Derived from the Greek term “ση’ψις” which means putrefaction, sepsis is the most common cause of death in intensive care unit (ICU) patients [1]. It is defined as a syndrome that involves biochemical, physiological and pathological abnormalities caused by an infection [2]. More recently, sepsis was defined in a consensus report published in 1992 and approved in 2003 as the systemic inflammatory response syndrome (SIRS) to infection [3]. The third and latest definition of sepsis by the Society of Critical Care Medicine’s 45th Critical Care Congress in 2016 was “a life-threatening organ dysfunction caused by a host’s dysfunctional response to infection (sepsis-3)” [4]. This definition of septic shock was updated to include the severe cellular/metabolic and circulatory disorder caused due to sepsis in addition to the host’s dysregulated inflammatory response, which increases mortality more than that from sepsis alone [1,2].

The incidence of sepsis has been increasing and is mainly dependent on the infecting organism; the causative organism in 48% of adults and 56% of children with sepsis and severe sepsis were viruses (29%), bacteria (27%) and parasites (1%). Viruses involved in sepsis are dengue virus, influenza viruses, rhinovirus, respiratory syncytial virus, rotavirus, adenovirus, norovirus and hantavirus. Gram-negative bacteria (*Escherichia coli*, *Klebsiella pneumoniae*, *Acinetobacter* spp., *Enterobacter* spp. and *Burkholderia pseudomallei*), and Gram-positive bacteria (*Staphylococcus aureus*, *Streptococcus pneumoniae*, *Streptococcus suis* and *beta-haemolytic Streptococcus* spp.) are the main pathogens which cause sepsis in both adults and children. Entamoeba histolytica, strongyloides and cryptosporidium are the main parasites responsible for sepsis [5]. A recent study reports that *Escherichia coli,* *Staphylococcus aureus* and *Klebsiella species* are the most commonly reported pathogens in early-onset sepsis [EOS], which occurs in the first 72 h of life, whereas *Staphylococcus aureus*, *Streptococcus pyogenes* and *Streptococcus pneumoniae* are the most commonly reported in late-onset sepsis [LOS], which occurs beyond 72 h of life in the developing countries of South Asia, Central Asia, East Asia, Pacific, Africa, Middle East and Latin America [6]. Another study found *Staphylococcus aureus*, methicillin-resistant *Staphylococcus aureus* (MRSA) and *Escherichia coli* in sepsis patients in Pakistan [7].

The pathogenesis of sepsis includes immune dysfunction, neuroendocrine-immune network abnormalities, imbalance in the inflammatory response, mitochondrial damage, endoplasmic reticulum stress, autophagy, coagulopathy and other pathophysiological processes at the molecular and cellular levels, which eventually lead to organ dysfunction, causing high mortality [2,8]. The conventional treatment for sepsis involves antibiotics, plasma, oxygen and other blood products to help with thromboembolic complications, and vasoactive medications and intravenous fluids for stabilizing the blood circulation and maintaining blood pressure [9]. There have been various sepsis studies in the Southeast Asia including Bangladesh [10,11], India [11,12,13], Indonesia [14,15], Nepal [6,11,16,17], Pakistan [7], Sri Lanka [18,19], Thailand [14,20] and Vietnam [14,21]. Appropriate management of sepsis is required, especially in Southeast Asian countries where the mortality rate is high [9]. This review focuses on the challenges encountered in the management of sepsis with reference to the diagnosis and time associated with sepsis, chloramphenicol boon over colistin resistance, antibiotic abuse, resource constraints and association of sepsis with COVID-19.

## 2. Methodology

An extensive literature search was conducted for articles on the sepsis management in Southeast Asia, using the keywords—“sepsis in Southeast Asia”, “septic shock”, “sepsis management”, “blood purification”, “CytoSorb^®^”, “antibiotic resistance in sepsis”, “colistin resistance”, “chloramphenicol in sepsis”, “antibiotic abuse”, “Personalized approach”, “COVID-19”, that were in PubMed, MEDLINE, Google Scholar or Science Direct and with filters “English language” and “full-text articles” (review articles, case reports, randomized controlled trials). Only articles published in peer-reviewed and indexed journals were selected; abstracts were excluded.

## 3. Epidemiological Data Associated with Sepsis Patients in Southeast Asia

**Figure 1:**
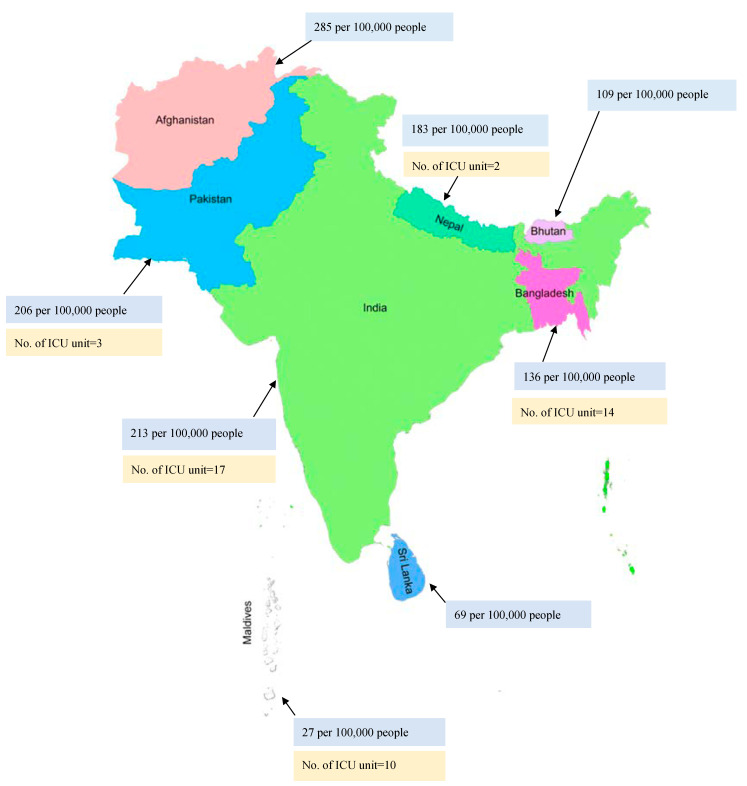
**Blue:** Mortality rate due to sepsis; **Yellow**: Prevalence of ICU units in various Southeast Asian countries.

The reported incidence of sepsis cases has been rising over the years. Approximately 48.9 million cases of sepsis were reported, with 19.7% of all global deaths due to sepsis [22]. High-income countries reported a higher incidence of sepsis, with about 2.8 million deaths per year [23]. There are over 970,000 cases of sepsis admitted to hospitals, with 50% deaths annually in the United States of America (USA) [6,24]. However, limited studies on sepsis are available from Southeast Asia. A recent study conducted in this region reported that 48% of adults and 56% of children were affected with sepsis and severe sepsis [5]. Overall, the mortality rate in Nepal and Bangladesh was reported to be around 39.3% and 37%, respectively, from sepsis [10,17]. The INDICAP study reported that 65% of ICU patients in India had sepsis with a 25% mortality rate, making India the Southeast Asian country with the second-highest mortality due to sepsis [25,26]. The mortality rate due to sepsis in various Southeast Asian countries is presented in Figure 1 (blue).

In Asia and Africa, the primary cause of adult deaths (90%) is lower respiratory tract infections, whereas, in infants and neonates, 70% of deaths are due to chest infections associated with sepsis [3]. The most frequent clinical presentation in patients with sepsis was reported to be acute respiratory infection (children: 63%, adults: 53%). Pneumonia was diagnosed as the most frequent respiratory tract infection in children (37%) as well as in adults (27%) [5]. Another study, including 69 patients with severe sepsis and septic shock, reported that the common origins of infection were abdomen (43.5%), central nervous system (CNS) (21.7%), urogenital tract (17.4%), respiratory tract (14.5%) and gastrointestinal tract (2.9%) [27]. The high mortality rate due to sepsis in developing countries might be due to the poorly understood epidemiology of sepsis, high prevalence of immunocompromised patients (HIV/AIDS) and lack of adequate facilities for treatment.

## 4. Clinical Investigation of Sepsis Patients in Southeast Asia

In 2001, the sepsis-2 definition required meeting the SIRS criteria along with confirmed or suspected infection. That is why SIRS criteria were taken as the standard for clinical sepsis diagnosis, which included two or more of either; heart rate above 90 beats per minute or temperature greater than 38 °C or less than 36 °C, white blood cell count greater than 12,000/μL or less than 4000/μL, or respiratory rate greater than 20 per minute [28]. In 2016, sepsis and severe sepsis were redefined and replaced SIRS in the Third International Sepsis Consensus Task Force definition [29], wherein the Sequential Organ Failure Assessment (SOFA) was recommended for clinical use to assess organ dysfunction. There are various factors included in the SOFA score: respiration (arterial oxygen partial pressure to fractional inspired oxygen), liver function (bilirubin levels), central nervous system function (Glasgow Coma Scale—GCS), cardiovascular function (mean arterial pressure), renal function (creatinine and urine output) and coagulation (platelet count) [28]. A wide variety of clinical tools and variables are used for early recognition of sepsis according to 2021 guidelines which include SIRS criteria, signs of infection, other vital signs, SOFA or qSOFA criteria, Modified Early Warning Score (MEWS) or National Early Warning Score (NEWS) [30]. A recent study conducted in Indonesia, Thailand, Vietnam and Pakistan reported that SOFA scores were quite helpful in predicting mortality rate in sepsis patients [14,31].

A rapid alternative to SOFA is a quick SOFA (qSOFA), which uses only a subset of this scoring system, including: altered mentation (GCS of 13 or less), a respiratory rate of 22 per minute or greater and a systolic blood pressure of below 100 mm Hg [28]. A Sri Lankan study reported qSOFA as a useful scoring system, especially in a resource-limited setup, as it does not need advanced monitoring [18].

In general, SOFA has shown a greater prognostic accuracy as compared to both SIRS and qSOFA [28]. An observational study conducted in Northeast Thailand over a 4-year study period included 4989 patients with severe malaria presenting as sepsis which utilized qSOFA, modified SOFA and 4 points of care (POC) diagnostics; whole blood lactate rapid diagnostic test (RDT), whole blood glucose RDT, pulse oximetry and the GCS. Among 153 malaria-confirmed patients, 75% had a qSOFA of ≥2 and had a weak correlation with malaria severity. However, the modified SOFA (score organ dysfunction and was calculated as the sum of coagulation, respiratory, cardiovascular, liver, renal and central nervous system parameters within 24 h of screening) had a strong correlation with malaria severity. All the 80 severe malaria patients who had a modified SOFA score of ≥2 were classified as sepsis positive, showing 100% sensitivity [20].

Additionally, there are various POC technologies for the diagnosis of sepsis based on biomolecular analysis, biomarkers of sepsis-like nucleic acids, proteins, pathogens or microbes and human cells. These technologies include lateral flow, dipstick, microfluidics and smartphone-enabled POC [28].

## 5. Time Factor Associated with Sepsis Patients in Southeast Asia

Sepsis patients with a relatively mild infection can deteriorate rapidly, requiring timely intervention. This transition from sepsis to septic shock results in increased morbidity and mortality [32]. Therefore, it is critical that the performance indicators for sepsis be formalized for early recognition to improve the condition of severe infection in children and adults [33,34]. In the case of adults, a set of guidelines have been devised by the UK Sepsis Six targets or Surviving Sepsis Campaign targets in which mentioned time for microbiological workups such as blood culture and antibiotics are within 1 h of the patient’s arrival [35]. A recent study reported that patients who were administered antibiotics between 3 h to 12 h of presentation had a 14% higher chance of in-hospital death than those who were administered antibiotics within 3 h [32]. Ekman and co-workers have reported that EOS treatment in infants is empirical, i.e., within 72 h of birth, based on a study conducted on almost 9000 infants in Nepal [36]. Another study conducted in India demonstrated that mortality due to EOS was 1.4 times higher than LOS [12]. In sepsis and septic shock, reliable data about the time to diagnosis, treatment and recovery in Southeast Asia is limited; however, timely intervention and proper diagnostics play a significant role in the proper management of sepsis.

## 6. Chloramphenicol Advantage over Colistin Resistance in Sepsis Patients and Antibiotic Abuse in Southeast Asia

Despite improving health and social conditions worldwide, there has been a spread of multidrug-resistant (MDR) organisms due to the overuse of antibiotics [37]. Inappropriate use of antibiotics increases the number of MDR bacteria as well as the rate of infection associated with mortality [38,39]. The mortality rate in septic shock and severe sepsis patients continues to increase due to inappropriate initial antimicrobial therapy [40].

**Figure 2:**
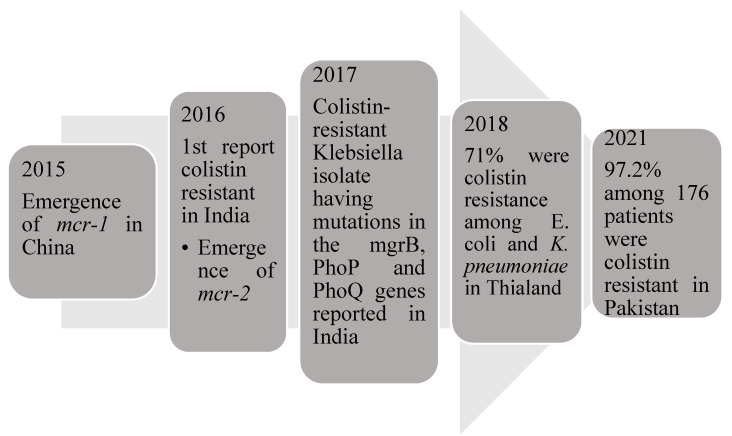
Emergence of colistin resistant gene and the cases associated with them.

Colistin, a polypeptide, has been used for treating critically-ill patients with Gram-negative sepsis. The emergence of the gene mcr-1 (mobilized colistin resistance) led to colistin resistance in the US, China, Germany and some European countries [41]. Qureshi et al. reported 20 patients with colistin-resistant *Acinetobacter baumannii*, where they were treated with a combination of Colistin methanesulfonate, carbapenem and ampicillin-sulbactam [42]. A previous study found that the rate of colistin resistance was 36.1% in 97 patients with isolates of carbapenem-resistant *Klebsiella pneumoniae*. Another study conducted in Greece reported that among 31 septic patients, 13 patients were colistin-resistant [43]. Currently, colistin is available in low- and middle-income countries as it is less expensive for use in most of the susceptible carbapenem-resistant organisms [44]; hence, it has become resistant in Southeast Asia. Kumar et al. were the first ones to identify colistin resistance in India due to the mcr1 gene [13]. Another study reported that colistin-resistant *Klebsiella* isolate had mutations in the mgrB, PhoP and PhoQ genes [45]. Pakistani and East Indian studies have reported colistin resistance due to the high use of meropenem and colistin in patients with sepsis [7,41]. Another study demonstrated that the prevalence of colistin resistance among *Escherichia coli* and *Klebsiella pneumoniae* isolates was very high [46]. A study conducted to determine colistin resistance in Gram-negative isolates showed 24 colistin-resistance isolates among the 94 MDR isolates and 6 colistin-resistant among 9 *Klebsiella pneumoniae* isolates [47]. Among the antibiotics tested for sensitivity in the study, tigecycline [75%] had good susceptibility, followed by chloramphenicol (62.5%); a combination therapy of chloramphenicol, tigecycline and fosfomycin had shown improvement in 25% patients [48]. Unfortunately, the increasing use of colistin to treat carbapenem-resistant bacteria has resulted in colistin resistance among Gram-negative bacteria, which are considered extensive drug-resistant (XDR) Gram-negative bacteria [49]. The emergence of colistin-resistant bacteria is mentioned in Figure 2.

Chloramphenicol is a semi-synthetic, broad-spectrum antibiotic derived from *Streptomyces venezuelae*. Chloramphenicol is highly efficacious in the treatment of sepsis associated with meningitis caused by Gram-positive bacteria such as *Haemophilus influenzae*, *Streptococcus pneumoniae* and *Neisseria meningitidis*. It is bactericidal at clinically achievable concentrations for Gram-positive bacteria whereas bacteriostatic for Gram-negative bacilli such as Enterobacteriaceae and *Staphylococcus aureus* [50]. A study reported the activity of chloramphenicol and several other antibiotics against 81 CRE isolates and showed that chloramphenicol, nitrofurantoin and ciprofloxacin had inhibitory activity against only 15–25% of the isolates [51]. However, it has been used for the treatment of sepsis in Southeast Asia, and resistance to this drug has rarely been reported [52]. A study conducted in Nepal reported that Gram-negative organisms were non-resistant to chloramphenicol and tigecycline showed [16]. An Indian study demonstrated that the majority of 33 *Klebsiella pneumoniae* and *E. coli* isolates were non-resistant to meropenem, amikacin, chloramphenicol and ciprofloxacin [7]. Chloramphenicol is a safe alternative. A comparison of minimal inhibitory concentration (MIC) of chloramphenicol with other drugs against various Gram-negative bacteria is discussed in Table 1.

The main reason for antibiotic resistance is the overuse of antibiotics to treat a number of infections. Belief in the general applicability of these antibiotics resulted in their excessive use, which led to the increased rate of antimicrobial resistance (AMR) [58]. Southeast Asia and the Middle East have a high level of AMR mainly because antibiotics are easily available over the counter (OTC) [38]. While antibiotic resistance occurs naturally, overuse or misuse of antibiotics in humans accelerates the process, as stated by the WHO [59]. Various epidemiological studies have reported a direct relationship between the intake of antibiotics and the emergence of resistant bacterial strains [60]. Zhu et al. found that the incidence of EOS in infants whose mothers were treated with antepartum antibiotics significantly increased from 2012 to 2018 [61].

A study conducted from 2000 to 2015 reported that antibiotic consumption has increased by 65% in low- and middle-income countries. [62]. Several studies proposed that the majority of antibiotic abuse occurs in a society where antibiotics are easily available as OTC drugs, such as low- and middle-income countries like Bangladesh, India and Thailand [11,63,64,65,66,67]. Hence, easy access to antibiotics and self-medication has become the primary concern for the overuse of antibiotics in society.

Sometimes, doctors prescribe several antibiotics in a life-threatening situation to reduce mortality, as an accurate diagnosis of infection takes time. Hence, the empirical use by doctors is the primary source of overuse of antibiotics [52]. In addition, there is a serial administration of antibiotics for the treatment of acutely ill patients by general practitioners (GP) based on experience without testing pathogen sensitivity [68]. This could be effective in curing the infectious disease if GP has guessed it correctly, but often antibiotic prescription is not appropriate for initial diagnosis. Hence, a repetitive course of different antibiotics is required until an effective treatment is established. Some GPs give an antibiotic prescription for viral infections such as respiratory tract infections due to similar symptoms to that of bacterial infections resulting in the emergence of antibiotic resistance [69]. A recent study conducted in low- and middle-income countries concluded that antibiotics are highly prescribed in primary care [70]. Various studies have even warned against the overuse of antibiotics as they are generally overprescribed [71,72]. Similarly, the use of antibiotics in farm animals’ feed without veterinary prescription has resulted in the passage of resistant bacteria from livestock to humans [73].

Serum procalcitonin (PCT) can be used as a guiding tool by the clinician to evaluate the therapy of sepsis and guide the antibiotic usage to avoid antibiotic abuse. An observational study on 98 patients with sepsis reported that PCT-guided therapy helps in reducing the duration of antibiotic usage [74]. A recent study divided the surgical intensive care patients with severe sepsis into two groups, control and PCT guided. In the PCT-guided group, the antibiotic treatment was discontinued when the level of PCT decreased to <35% of the initial value. This study observed that the PCT-guided algorithm reduced the expense of the treatment as well as the use of antibiotics [75]. Another therapy evaluated PCT-guided antibiotic therapy compared to standard therapy in chronic obstructive pulmonary disease patients. PCT-guided therapy reduced antibiotic exposure as compared to the standard therapy [76].

## 7. Resource Constraints about High Population and Limitations of Funds, Personnel, Infrastructure, etc. Sepsis Patients in Southeast Asia

There are only 1.7% of all biomedical research publications related to critical care in low- and middle-income countries; however, the need for critical care research is huge [77]. Surviving Sepsis Campaign (SSC) developed guidelines to reduce mortality in high-income countries; however, the effectiveness of these guidelines requires more assessment in low- or middle-income countries [78]. Therefore, there is an urgent need to comprehend the cost-effectiveness, benefits and resource-limited settings with poor ICU capacity for the execution of sepsis care intervention in these countries [79].

Hung et al. reported that very important barriers to optimal care of sepsis patients were inadequate nursing human resources (50%) followed by doctors’ workload (41.7%) [35]. An Asian study reported that there are less than three ICU beds per 100,000 persons in most low- and middle-income countries [80]. An observational study conducted in Thailand on sepsis patients reported that the majority of the sepsis patients were managed in the general wards due to a lack of ICU resources [81]. Another study reported a lack of infrastructure, such as a central pipeline for oxygen, in most of the government-funded hospitals in Nepal. As recommended by the World Health Organization (WHO), there should be 2.3 doctors per 1000 persons; however, this study reported that only 1 doctor was available per 1000 persons in Nepal [82]. The limited number of ICU units in various countries of Southeast Asia is presented in Figure 1 (yellow).

Moreover, health expenditure as a share of gross domestic product (GDP) is just 2.1%; hence, the management of sepsis in low- and middle-income countries has further challenges for clinicians [83]. In India, a recent study reported that about 10–20% of emergency care children are referred to hospital, but there is late presentation, delay in recognition, a lack of resources and illness severity in the first 24 h of hospitalization, with almost every third patient dying in this period [84]. Though improving oxygen therapy in resource-limited settings has been shown to reduce mortality in sepsis [85], these respiratory supports are expensive and need a high level of maintenance and technical expertise for proper functioning. An Indian study observed that the major barriers in managing infections included the need for a high number of nursing staff, time spent on training new staff, heavy clinical workload and limitations in language competency [86]. Several low- and middle-income countries lack the funds, expertise or infrastructure to provide such technology to all the patients [84]. A recent study reported that patients in Vietnam are struggling to get either enough resources or adequate diagnostic facilities or proper treatment for sepsis in both local and central settings. Further, initiation of treatment of sepsis generally gets delayed, including the administration of antibiotics [21]. Early use of norepinephrine has shown its benefits in the control of sepsis in resource-limited regions. The Phase-II randomized trial on the early use of a low dose of vasopressors for septic shock has shown beneficial effects in clinical resuscitation. Bima et al. have reported an improvement in mortality rate by the use of norepinephrine in a low-resource setting outside ICU [87,88]. Some more national strategies such as this are required, especially in resource-constrained countries, to combat problems related to sepsis.

## 8. Sepsis and COVID-19 in Southeast Asia

In March 2020, Coronavirus 2019 (COVID-19) or severe acute respiratory syndrome coronavirus-2 (SARS-CoV-2) was declared a pandemic by the WHO [89]. It has affected healthcare, economic, social and environmental pathways worldwide. The second wave has evolved drastically as compared to the first wave, due to which the number of cases tremendously increased [90]. The total number of COVID-19-positive cases in India, Bangladesh, Nepal and Sri Lanka is 43.16 million, 19.54 million, 0.98 million and 0.66 million, respectively, as of 2 June 2022 [91]. The rise in COVID-19 patients at a phenomenal speed during 2020 and 2021 led to the exhaustion of the workforce as well as resources, especially in low- and middle-income countries. Further, studies have reported that there was an acute shortage of oxygen supply, ventilators, hospital beds and medicines for COVID-19 patients in these countries during the pandemic situation [13].

COVID-19 is known to be associated with sepsis, acute respiratory distress syndrome (ARDS), cytokine release syndrome (CRS), thromboembolic disease and multi-system organ failure (MSOF) [92]. Majority of patients require ICU, which has challenged the healthcare system worldwide. The requirement for mechanical ventilator or ICU admission associated with COVID-19 and sepsis is as high as 39 to 72% [93]. About 5% of the patients with mild or moderate COVID-19 required organ support [94].

Immune dysregulation is usually observed in COVID-19 patients causing organ damage and cytokine storm [95]. The cytokine profile in COVID-19 is explained by increasing interleukin IL-2, IL-6, IL-7, interferon-γ (INF-γ) inducible protein 10, granulocyte colony-stimulating factor (GCS-F) and tumor necrosis factor-α (TNF-α) [89]. A recent study has reported that approximately 28% of patients died due to COVID-19 associated with cytokine storm and sepsis [96]. COVID-19 patients associated with sepsis can have a poor prognosis due to co-infection, increasing the high risk in the population; especially elderly patients. A previous retrospective multicenter study demonstrated that 50% of the patients who died of COVID-19 tend to develop secondary bacterial co-infection during the course of hospitalization [97]. In the general population, the mortality rate due to COVID-19 ranges from 1.4 to 8%, and it significantly increases the number of patients who require ICU admission [98].

## 9. Recommendations by the Authors

A panel of five expert doctors from different regions of Southeast Asia, namely Bangladesh, India, Nepal and Sri Lanka, was convened on 22 September 2021 to review the existing literature on the management of sepsis in their respective countries. The following were the recommendation by the experts:

### 9.1. Data Collection and Sharing

Sepsis is a multifaceted disease with a wide variation in causative microorganisms, sepsis rate and outcome in patients. The Southeast Asia region lacks proper sanitation, clean water, hygienic infrastructure, funds and personnel to attend to the patients and has a dense population, which increases the risk of the emergence and spread of sepsis [99]. Structured data collection on clinical, biochemical and outcome parameters should be established. Online platforms for discussions such as WhatsApp groups of medical students and physicians from India, Nepal, Bangladesh, Maldives, USA, UK, Canada, France and Australia are already functional. On this platform, these countries regularly collect and discuss patient records and data in the hope of obtaining online feedback through conversational engagement with other colleagues of this group. This is generally supported by the current best evidence support as case-based blended learning ecosystems [100]. However, the spread of misinformation through these communication platforms is still a concern, leading to the implementation of prevention policies for the spread of misinformation by various stakeholders. Likewise, more data from various countries should be pooled and published, which might be helpful to all other clinicians, medical students and even the general public for sepsis management.

### 9.2. Personalized Approach to Sepsis Management

Sepsis is a complex disease characterized by a different inflammatory response in every patient of sepsis. The most successful way to facilitate the treatment of sepsis is through a personalized approach, as individual patients have a unique profile of immune activation against particular pathogens [101]. A personalized approach is used to prevent, diagnose and treat individual patient characteristics and is based on “omics” based data (genomics, transcriptomics, proteomics, metabolomics, epigenomics, pharmacogenomics, interactomics and microbiomic). Genome-wide association studies and genomic signatures have the potential to recognize genetic variants that could respond to specific immunomodulatory interventions through the identification of alterations of specific pathways that can be addressed by pro-inflammatory or anti-inflammatory cytokines. This treatment is frequent in the developed countries’ healthcare systems, including the USA, UK and the European Union (EU). Although many developing countries in Southeast Asia have also started adopting this treatment strategy for various diseases, for sepsis treatment, the personalized medicine approach has not been reported in these countries to date [102]. A recent study reported that the development of metabolomics associated with sepsis might have a more general impact on the healthcare system over the world. For instance, there are a limited number of new antimicrobial agents for an increasing number of antimicrobial-resistant pathogens; hence, metabolic fingerprinting applicability complements our knowledge and suggested drug discovery [103]. As previously mentioned, the case-based blended learning ecosystems also help bridge the gap between age-old precision approaches with modern technology and omics-driven approaches between developed and developing countries. It is a practical tool to study the case-based description in both high- and low-resource settings concerning personalized medicine. This description further illustrates the patient’s journey from “age-old precision thinking”, in low-resource settings, progressing to “omics-driven” high-resource settings regarding precision medicine [104]. Another study by Ray and Goyal reported that increasing the use of rapid nucleic acid sequencing, along with proteomic-, epigenomic- and metabolomic-based tools to determine the molecular variability in host response, is promising for early diagnosis and personalized treatment in sepsis in India [100].

### 9.3. Conventional Approach to Sepsis Management

Early antibiotics administration, fluid resuscitation for treatment of hypoperfusion and vasopressor application are recommendations proposed by SSC to reduce sepsis-related mortality [1]. These approaches work well in resource-constrained regions such as Southeast Asia. Antibiotics should be given as soon as possible after a diagnosis of sepsis to reduce the risk of mortality. However, empiric and appropriate antibiotic administration is also important in addition to timely intervention [8]. The majority of antibiotics administered during sepsis, such as piperacillin/tazobactam, ceftriaxone, cefepime, meropenem, and imipenem/cilastatin, have effectiveness against Gram-positive organisms, such as methicillin-susceptible *Staphylococcus aureus*, or MSSA, and Streptococcal species. Ceftriaxone, ampicillin/sulbactam and ertapenem are considered in sepsis related to community-acquired intra-abdominal or urinary tract infections. Aztreonam, aminoglycosides and ciprofloxacin are used in patients having a severe penicillin allergy. However, these agents are ineffective against Gram-positive bacteria; therefore, vancomycin or linezolid are added along with these agents. Information about prior antibiotics exposure is a must in the proper management of sepsis. Narrower regimens are advised in patients showing rapid response to fluid replacement and patients who do not need vasopressor. Combination antibiotic therapy consisting of beta-lactam agent plus aminoglycoside or quinolone can be considered in severely ill patients; however, various studies have reported no advantage of combination therapy in preventing antibiotic resistance and improvement in patient condition [105].

The main treatment target in sepsis is to regulate the blood volume. Fluid resuscitation is performed to maintain sufficient perfusion in tissues. Energetic fluid intake for rapid restoration of tissue perfusion, normal heart rate and arterial blood pressure is performed in sepsis patients. Volume is administered with crystalloid and total volume varies depending on the condition of the patient [1]. Vasopressor requirement and its advantage in reducing mortality in sepsis patients of resource-limited Southeast Asian countries have been observed by various studies as described in Section 7 [87,88]. The administration of hydrocortisone or prednisolone in patients requiring catecholamines is also recommended for the management of sepsis in resource-limited regions [106].

### 9.4. Innovative Therapeutic Alternatives

**Figure 3:**
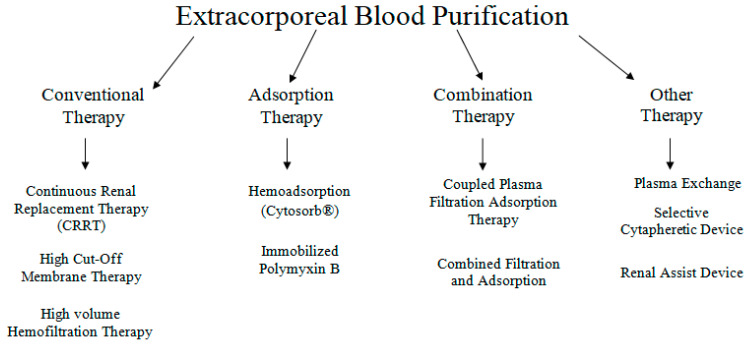
Various available extracorporeal therapies.

In sepsis, extracorporeal blood purification techniques (ECT) have been proposed as an adjunctive therapy, based on the concept that removing bacterial toxins or pro- and anti-inflammatory mediators could attenuate the sepsis-related inflammatory response and hence limit organ damage [107]. Different types of ECTs are presented in Figure 3. ECT’s work on the principle of extracorporeal blood purification by using an extracorporeal blood circuit and a special adsorber that clears the blood prior to being re-administered to the patient [108]. CytoSorb^®^ (CytoSorbents Corporation, Monmouth Junction, NJ, USA) is the first specially approved extracorporeal cytokine hemadsorber in the EU and is approved to remove elevated levels of cytokines, bilirubin and myoglobin along with drugs like ticagrelor and rivaroxaban, which are fundamental in managing critically ill patients and high-risk surgery patients [109].

CytoSorb^®^ is a novel acute hemadsorption technique with proven clinical pieces of evidence based on different types of studies for the removal of hydrophobic middle-high sized inflammatory mediators to stabilize hemodynamics. It is the most widely studied hemadsorption treatment that improves survival rates and reduces the ICU stay of patients. In resource-constrained countries, CytoSorb^®^ therapy in conjunction with a personalized approach, can improve the outcome by optimizing the therapy for individual sepsis patients. The authors’ extensive positive clinical experience and agreement on the CytoSorb^®^ approach have necessitated a detailed discussion of CytoSorb^®^ therapy.

### 9.5. CytoSorb^®^ Therapy: Optimal Dosage and Early Initiation Improves Clinical Outcome

CytoSorb^®^ (CytoSorbents Corporation, Monmouth Junction, NJ, USA) is CE Mark approved under the Medical Devices Directive, is ISO 10993 biocompatible and is manufactured in the US under ISO 13485 certification [110]. It has been recently used to treat over 7000 critically ill patients infected with COVID-19 in 30 countries [111]. CytoSorb^®^ technology is a hemoperfusion sorbent cartridge characterized by a resin with polymer beads, through a combination of hydrophobic interactions and size exclusion, allowing the absorption of pro- and anti-inflammatory cytokines [112]. CytoSorb^®^ has been shown to improve survival in septic shock patients, provided that the applied dose is high enough and for an optimal duration of time [113]. As of 2022, more than 170,000 CytoSorb^®^ treatments have been used in more than 800 hospitals all over the world, and its usage has been proven to be safe and well-tolerated in patients [114]. Various studies have shown the efficacy and safety of CytoSorb^®^ for the treatment of patients with sepsis and sepsis shock [115,116].

CytoSorb^®^ is approved as Emergency Use Authorization (EUA) by the Food and Drug Administration (FDA) and Drugs Controller General of India (DCGI) for COVID-19 patients [117]. Several clinical trials are ongoing worldwide to investigate the effects of CytoSorb^®^ in COVID-19 patients [118]. In a recent study, 50 patients with COVID-19 and associated sepsis, ARDS, acute kidney injury and hyper inflammation were treated using CytoSorb^®^ with continuous renal replacement therapy (CRRT). Among them, 35 patients survived, demonstrating decreased SOFA score, IL-6, C-reactive protein and D-dimers after treatment with CytoSorb^®^ with CRRT [119]. Mehta et al. reported a case series of severely ill COVID-19 patients admitted to ICU treated with CytoSorb^®^ therapy. The patients showed significant improvement in clinical outcomes and biochemical parameters post-CytoSorb^®^ therapy. The mean arterial pressure (MAP) improved, C-reactive protein levels decreased post-therapy, and the patients were discharged [120]. Song et al. reported the high survival rates of critically-ill COVID-19 patients on extracorporeal membrane oxygenation by using CytoSorb^®^ therapy in the retrospective multi-centric study [121]. Table 2 summarizes the peer-reviewed articles examining the benefits of CytoSorb^®^ treatment [115,116,120,122,123,124,125,126,127,128,129,130,131,132,133,134,135,136,137].

Most of the evidence regarding the effectiveness of CytoSorb^®^ is based on case series, case studies and retrospective reports. A randomized controlled pilot study of 20 septic shock patients with no need for renal replacement therapy concluded that vasopressor needs, procalcitonin (PCT) and big-endothelin-1 were reduced by CytoSorb^®^ treatment [138]. A randomized controlled trial on sepsis patients reported significant removal of IL-6 in pre- and post-adsorbers measurements; however, no significant reduction in systemic IL-6 levels by CytoSorb^®^ therapy was found. This non-significant reduction in IL-6 levels was due to the inhomogenous distribution of patients between control and CytoSorb^®^ groups. Moreover, this study was just focused on confirming the safety and efficacy of CytoSorb^®^, which it has demonstrated well [139]. In addition, randomized controlled trials pose practical challenges, such as the comparability of both groups becoming an issue in critically-ill patients. The majority of randomized controlled trials have proven to show no effect of the tested intervention on outcomes in last 3 decades, wasting both time and money. Instead of randomized controlled trials, real-world evidence can benefit in determining the potential of CytoSorb^®^ in the management of sepsis. Moreover, septic shock is a heterogenous phenotype; thus, objective evaluation of CytoSorb^®^ is difficult in septic shock. CytoSorb^®^ is not used to treat any type of disease; it is a device to remove harmful inflammatory cytokines from the body, making it a promising approach for the management of sepsis [140].

## 10. Conclusions

Sepsis and septic shock account for high morbidity and mortality worldwide in general and particularly in Southeast Asian countries due to several reasons. Global epidemiological data helps to provide crucial information for future healthcare planning and increases awareness of sepsis and septic shock; however, limited data is available in Southeast Asian countries related to the time factor, chloramphenicol boon, antibiotics abuse and resource limitation. Therefore, consolidation and systematic harvesting of data become indispensable for healthcare professionals and policy makers to manage the burden of sepsis efficiently. In addition, it is now well established that absence of evidence is not evidence in absence. Therefore, adopting a personalized treatment approach wherever and whenever desirable and embracing novel extracorporeal blood purification technologies could further enhance patient outcomes and alleviate the burden of sepsis.

## Figures and Tables

**Table 1:** Comparison of MIC values [mg/L] of various antibiotics with Chloramphenicol.

Organisms	*Klebsiella pneumonia*	*Escherichia coli*	*Staphylococcus aureus*
Chloramphenicol	4–256	0.015–10,000	0.200–256
Ciprofloxacin	0.25–0.5	15–300	0.09–64.00
Vancomycin	256–1024	64–1024	0.5–3.0
Tigecycline	50–200	0.03–4	0.016–0.47
Meropenem	4000–16,000	0.015–0.25	0.12–3.00
Amikacin	320–256 00	2–16	0.500–8.00

Refs. [53,54,55,56,57].

**Table 2:** Peer-reviewed studies showing the effectiveness of CytoSorb^®^ treatment.

S. No.	No. of Patients	Study Type	Comorbidities/Indication of CytoSorb^®^	Clinical Outcomes
1.	198	Retrospective control study	Septic shock	Early start of CytoSorb^®^ therapy significantly improved the survival of septic shock patients. A dynamic scoring system to assess the efficacy of CytoSorb^®^ therapy was also developed.
2.	116	Retrospective study	Septic shock	Improvement in 28-day survival, both on the basis of observed versus predicted mortality rates, was observed in CytoSorb^®^ + CRRT group as compared CRRT alone group.
3.	100	Observational and retrospective study	Sepsis and septic shock.	Survivors (*n* = 40) Non-survivors (*n* = 60)
4.	84 (Group 1: CytoSorb + CRRT, 42; Group 2: CRRT)	Retrospective genetic matched control study	Septic Shock	Catecholamines levels were reduced to half within 24 h after initiation of CytoSorb therapy. In hospital and 28-day mortality were reduced in CytoSorb^®^ group
5.	45	Observational multicenter study	Sepsis and septic shock.	Mortality rate: 48.8% after CytoSorb^®^ therapy. 75% survival rate in patients given treatment in <24 h of ICU admission and 68% survival rates within 24–48 h of ICU admission
6.	36	Observational and retrospective study	Sepsis and septic shock	Procalcitonin and total leucocyte count was reduced within 24 h of initiation of therapy. Sepsis related SOFA score was reduced. Survivors (17) Non-survivors (*n* = 19)
7.	26	Retrospective case series	COVID-19 and acute respiratory distress syndrome	Significant reductions in norepinephrine, and inflammatory markers, with improvements in respiratory and other organ functions by use of CytoSorb^®^.
8.	25 (Group 1: CRRT, 15; Group 2: CRRT + CytoSorb^®^)	Retrospective analysis	Multiorgan dysfunction syndrome	Mortality rate: 53.3% in Group I and 60.0% in Group II
9.	16 (CytoSorb^®^: 8; control: 8)	Retrospective pilot study	Elective major aortic surgery for aortic aneurysm and/or aortic dissection.	
10.	13	Retrospective case series	COVID-19, organ failure and acute respiratory distress syndrome	Significant reduction in inflammatory mediators (interleukin 6), hemodynamic stabilization with a concomitant decrease in requirements for vasoactive substances (norepinpethrine), and a pronounced improvement in lung function were observed with combined therapy of CRRT and CytoSorb^®^
11.	3 1	Case Series Case Study	Hypertension Respiratory failure type-1/Other Hypertension Diabetes/Septic with shock Septic shock with multi-organ dysfunction (MODS) and a low perfusion state with a history of diabetes mellitus type II, hypertension, obstructive sleep apnea, hypothyroidism and morbid obesity.	Significant improvement in biochemical parameters and mean arterial pressure was observed with reduction in C-reactive proteins. All patients survived
12.	1	Case Study	Septic shock with multi-organ dysfunction (MODS) and a low perfusion state with a history of diabetes mellitus type II, hypertension, obstructive sleep apnea, hypothyroidism and morbid obesity.	Reduction in lactate levels with improved clinical parameters were observed post-CytoSorb^®^ therapy. Vasopressor requirement was reduced to nil. Patient survived
13.	1	Case Study	Dengue haemorrhagic fever associated with SIRS, acute fulminant hepatic failure with encephalopathy and oliguria	Liver function tests i.e., SGOT, SGPT were improved with improved platelet count. Patient was hemodynamically stable during discharge.
14.	1	Case Study	Dengue fever with septic shock and multiorgan failure admitted in the intensive care.	Survived
15.	1	Case Study	SIRS and renal dysfunction after heart transplantation	Vasopressor was weaned completely post-CytoSorb therapy with reduction in serum lactate levels depicting clinical improvement in patient.
16.	1	Case Study	Acute Kidney Injury due to Rhabdomyolysis	Patient showed hemodynamic stability post-CytoSorb^®^ therapy and survived
17.	1	Case Study	Sepsis complicated by typhoid fever	Survived
18.	1	Case Report	Sepsis with multiple organ dysfunction syndrome with the manifestation of acute respiratory distress syndrome (ARDS) and acute renal failure (AKI)	Reduction in catecholamine demand with reduction in serum lactate levels. Patient survived
19.	1	Case report	Sepsis with multiple organ dysfunction syndrome	Survived

## Data Availability

Not applicable.

## References

[1] Rello J., Valenzuela-Sánchez F., Ruiz-Rodriguez M., Moyano S. (2017). Sepsis: A review of advances in management. Adv. Ther..

[2] Huang M., Cai S., Su J. (2019). The pathogenesis of sepsis and potential therapeutic targets. Int. J. Mol. Sci..

[3] Salomão R., Ferreira B.L., Salomão M.C., Santos S.S., Azevedo L.C.P., Brunialti M.K.C. (2019). Sepsis: Evolving concepts and challenges. Braz. J. Med. Biol. Res..

[4] Monard C., Rimmele T., Ronco C. (2019). Extracorporeal blood purification therapies for sepsis. Blood Purif..

[5] Research, Southeast Asia Infectious Disease Clinical (2017). Causes and outcomes of sepsis in southeast Asia: A multinational multicentre cross-sectional study. Lancet Glob. Health.

[6] Ansari S., Nepal H.P., Gautam R., Shrestha S., Neopane P., Chapagain M.L. (2015). Neonatal septicemia in Nepal: Early-onset versus late-onset. Int. J. Pediatr..

[7] Rehman Z.U., Shah M.H., Afridi M.N.S., Sardar H., Shiraz A. (2021). Bacterial Sepsis Pathogens and Resistance Patterns in a South Asian Tertiary Care Hospital. Cureus.

[8] Polat G., Ugan R.A., Cadirci E., Halici Z. (2017). Sepsis and septic shock: Current treatment strategies and new approaches. Eurasian J. Med..

[9] Bonjar M.R.S., Bonjar L.S. (2015). A prospective treatment for sepsis. Drug Des. Dev. Ther..

[10] Ahmed S., Applegate J.A., Mitra D.K., Callaghan-Koru J.A., Mousumi M., Khan A.M., Joarder T., Harrison M., Ahmed S., Begum N. (2019). Implementation research to support Bangladesh Ministry of Health and Family Welfare to implement its national guidelines for management of infections in young infants in two rural districts. J. Health Popul. Nutr..

[11] Haniffa R., Mukaka M., Munasinghe S.B., De Silva A.P., Jayasinghe K.S.A., Beane A., de Keizer N., Dondorp A.M. (2017). Simplified prognostic model for critically ill patients in resource limited settings in South Asia. Crit. Care.

[12] Meshram R.M., Gajimwar V.S., Bhongade S.D. (2019). Predictors of mortality in outborns with neonatal sepsis: A prospective observational study. Niger. Postgrad. Med. J..

[13] Godara S.M., Kute V.B., Trivedi H.L., Vanikar A.V., Shah P.R., Gumber M.R., Patel H.V., Gumber V.M. (2014). Clinical profile and outcome of acute kidney injury related to pregnancy in developing countries: A single-center study from India. Saudi J. Kidney Dis. Transplant..

[14] Lie K.C., Lau C.Y., Chau N.V.V., West T.E., Limmathurotsakul D. (2018). Utility of SOFA score, management and outcomes of sepsis in Southeast Asia: A multinational multicenter prospective observational study. J. Intensive Care.

[15] Pradipta I.S., Sodik D.C., Lestari K., Parwati I., Halimah E., Diantini A., Abdulah R. (2013). Antibiotic resistance in sepsis patients: Evaluation and recommendation of antibiotic use. N. Am. J. Med. Sci..

[16] Nepal D., Agrawal S., Shrestha S., Rayamajhi A. (2020). Bacteriological profile and antibiotic susceptibility pattern of Neonatal Septicemia in Kanti Children Hospital, Nepal. J. Gandaki Med. Coll.-Nepal.

[17] Ghimire R., Shakya Y.M., Shrestha T.M., Neupane R.P. (2020). The utility of red cell distribution width to predict mortality of septic patients in a tertiary hospital of Nepal. BMC Emerg. Med..

[18] Dassanayake V.G. (2016). Sepsis and septic shock: Can we win the battle against this hidden crisis?. Sri Lanka J. Surg..

[19] Matthias T., Ranasinghe T., Mallawaarachchi C., Wijekoon S., Indrakumar J. (2020). A study on adherence to surviving sepsis campaign bundle at a tertiary care hospital in Sri Lanka. Int. J. Infect. Dis..

[20] Teparrukkul P., Hantrakun V., Imwong M., Teerawattanasook N., Wongsuvan G., Day N.P., Dondorp A.M., West T.E., Limmathurotsakul D. (2019). Utility of qSOFA and modified SOFA in severe malaria presenting as sepsis. PLoS ONE.

[21] Do S.N., Luong C.Q., Pham D.T., Nguyen M.H., Nguyen N.T., Huynh D.Q., Hoang Q.T.A., Dao C.X., Le T.M., Bui H.N. (2021). Factors relating to mortality in septic patients in Vietnamese intensive care units from a subgroup analysis of MOSAICS II study. Sci. Rep..

[22] Rudd K.E., Johnson S.C., Agesa K.M., Shackelford K.A., Tsoi D., Kievlan D.R., Colombara D.V., Ikuta K.S., Kissoon N., Finfer S. (2020). Global, regional, and national sepsis incidence and mortality, 1990–2017: Analysis for the Global Burden of Disease Study. Lancet.

[23] Rieder M., Zahn T., Benk C., Lother A., Bode C., Staudacher D., Duerschmied D., Supady A. (2021). Cytokine adsorption in a patient with severe coronavirus disease 2019 related acute respiratory distress syndrome requiring extracorporeal membrane oxygenation therapy: A case report. Artif. Organs.

[24] Fan S.L., Miller N.S., Lee J., Remick D.G. (2016). Diagnosing sepsis–The role of laboratory medicine. Clin. Chim. Acta.

[25] Divatia J.V., Mehta Y., Govil D., Zirpe K., Amin P.R., Ramakrishnan N., Kapadia F.N., Sircar M., Sahu S., Bhattacharya P.K. (2021). Intensive Care in India in 2018–2019: The Second Indian Intensive Care Case Mix and Practice Patterns Study. Indian J. Crit. Care Med. Peer-Rev. Off. Publ. Indian Soc. Crit. Care Med..

[26] Kshirsagar A., Kale S., More S., Anturkar R., Vispute S. (2021). An Application of Predictive Analytics for Early Detection of Sepsis: An Overview. Int. J. Eng. Res. Technol..

[27] Sarker S.K., Azam M.S., Mondal M.K., Goswami U.K., Mohsin M. (2020). Demographic Profiles and Sources of Infection among Septic Patients admitted at ICU of a Public Hospital in Dhaka City. Bangladesh J. Infect. Dis..

[28] Oeschger T., McCloskey D., Kopparthy V., Singh A., Erickson D. (2019). Point of care technologies for sepsis diagnosis and treatment. Lab Chip.

[29] Singer M., Deutschman C.S., Seymour C.W., Shankar-Hari M., Annane D., Bauer M., Bellomo R., Bernard G.R., Chiche J.D., Coopersmith C.M. (2016). The third international consensus definitions for sepsis and septic shock [Sepsis-3]. JAMA.

[30] Evans L., Rhodes A., Alhazzani W., Antonelli M., Coopersmith C.M., French C., Machado F.R., Mcintyre L., Ostermann M., Prescott H.C. (2021). Surviving sepsis campaign: International guidelines for management of sepsis and septic shock 2021. Intensive Care Med..

[31] Baig M.A., Sheikh S., Hussain E., Bakhtawar S., Khan M.S., Mujtaba S., Waheed S. (2018). Comparison of qSOFA and SOFA score for predicting mortality in severe sepsis and septic shock patients in the emergency department of a low middle income country. Turk. J. Emerg. Med..

[32] Wu Y.P., Lauffenburger J.C. (2020). Effectiveness of corticosteroids in patients with sepsis or septic shock using the new third international consensus definitions [Sepsis-3]: A retrospective observational study. PLoS ONE.

[33] Machado F.R., Ferreira E.M., Schippers P., de Paula I.C., Saes L.S.V., de Oliveira F.I., Tuma P., Nogueira Filho W., Piza F., Guare S. (2017). Implementation of sepsis bundles in public hospitals in Brazil: A prospective study with heterogeneous results. Critical Care.

[34] Noritomi D.T., Ranzani O.T., Monteiro M.B., Ferreira E.M., Santos S.R., Leibel F., Machado F.R. (2014). Implementation of a multifaceted sepsis education program in an emerging country setting: Clinical outcomes and cost-effectiveness in a long-term follow-up study. Intensive Care Med..

[35] National Health Service UK Sepsis Guidance Implementation Advice for Adults. https://www.england.nhs.uk/wp-content/uploads/2017/09/sepsis-guidance-implementation-advice-for-adults.pdf.

[36] Ekman B., Paudel P., Basnet O., Ashish K.C., Wrammert J. (2020). Adherence to World Health Organisation guidelines for treatment of early onset neonatal sepsis in low-income settings; a cohort study in Nepal. BMC Infect. Dis..

[37] Busani S., Serafini G., Mantovani E., Venturelli C., Giannella M., Viale P., Mussini C., Cossarizza A., Girardis M. (2019). Mortality in patients with septic shock by multidrug resistant bacteria: Risk factors and impact of sepsis treatments. J. Intensive Care Med..

[38] Zilahi G., Artigas A., Martin-Loeches I. (2016). What’s new in multidrug-resistant pathogens in the ICU?. Ann. Intensive Care.

[39] Tosi M., Roat E., De Biasi S., Munari E., Venturelli S., Coloretti I., Biagioni E., Cossarizza A., Girardis M. (2018). Multidrug resistant bacteria in critically ill patients: A step further antibiotic therapy. J. Emerg. Crit. Care Med..

[40] Shorr A.F., Micek S.T., Welch E.C., Doherty J.A., Reichley R.M., Kollef M.H. (2011). Inappropriate antibiotic therapy in Gram-negative sepsis increases hospital length of stay. Crit. Care Med..

[41] Exner M., Bhattacharya S., Christiansen B., Gebel J., Goroncy-Bermes P., Hartemann P., Heeg P., Ilschner C., Kramer A., Larson E. (2017). Antibiotic resistance: What is so special about multidrug-resistant Gram-negative bacteria?. GMS Hyg. Infect. Control.

[42] Qureshi Z.A., Hittle L.E., O’Hara J.A., Rivera J.I., Syed A., Shields R.K., Pasculle A.W., Ernst R.K., Doi Y. (2015). Colistin-resistant *Acinetobacter baumannii*: Beyond carbapenem resistance. Clin. Infect. Dis..

[43] Papathanakos G., Andrianopoulos I., Papathanasiou A., Priavali E., Koulenti D., Koulouras V. (2020). Colistin-resistant *Acinetobacter baumannii* bacteremia: A serious threat for critically ill patients. Microorganisms.

[44] Ambreen G., Salat M.S., Hussain K., Raza S.S., Ali U., Azam I., Iqbal J., Fatmi Z. (2020). Efficacy of colistin in multidrug-resistant neonatal sepsis: Experience from a tertiary care center in Karachi, Pakistan. Arch. Dis. Child..

[45] Hamel M., Rolain J.M., Baron S.A. (2021). The History of Colistin Resistance Mechanisms in Bacteria: Progress and Challenges. Microorganisms.

[46] Eiamphungporn W., Yainoy S., Jumderm C., Tan-Arsuwongkul R., Tiengrim S., Thamlikitkul V. (2018). Prevalence of the colistin resistance gene mcr-1 in colistin-resistant *Escherichia coli* and *Klebsiella pneumoniae* isolated from humans in Thailand. J. Glob. Antimicrob. Resist..

[47] Ramesh N., Prasanth M., Ramkumar S., Suresh M., Tamhankar A.J., Gothandam K.M., Karthikeyan S., Bozdogan B. (2016). Colistin susceptibility of gram-negative clinical isolates from Tamil Nadu, India. Asian Biomed..

[48] Arjun R., Gopalakrishnan R., Nambi P.S., Kumar D.S., Madhumitha R., Ramasubramanian V. (2017). A study of 24 patients with colistin-resistant Gram-negative isolates in a tertiary care hospital in South India. Indian J. Crit. Care Med. Peer-Rev. Off. Publ. Indian Soc. Crit. Care Med..

[49] Bialvaei A.Z., Samadi Kafil H. (2015). Colistin, mechanisms and prevalence of resistance. Curr. Med. Res. Opin..

[50] Rahal J.J., Simberkoff M.S. (1979). Bacteriocidal and bacteriostatic action of chloramphenicol against meningeal pathogens. Antimicrob. Agents Chemother..

[51] Livermore D.M., Warner M., Mushtaq S., Doumith M., Zhang J., Woodford N. (2011). What remains against carbapenem-resistant Enterobacteriaceae? Evaluation of chloramphenicol, ciprofloxacin, colistin, fosfomycin, minocycline, nitrofurantoin, temocillin and tigecycline. Int. J. Antimicrob. Agents.

[52] Batty E.M., Cusack T.P., Thaipadungpanit J., Watthanaworawit W., Carrara V., Sihalath S., Hopkins J., Soeng S., Ling C., Turner P. (2020). The spread of chloramphenicol-resistant Neisseria meningitidis in Southeast Asia. Int. J. Infect. Dis..

[53] Fuursted K., Schumacher H. (2002). Significance of low-level resistance to ciprofloxacin in *Klebsiella pneumoniae* and the effect of increased dosage of ciprofloxacin in vivo using the rat granuloma pouch model. J. Antimicrob. Chemother..

[54] Sueke H., Kaye S., Neal T., Murphy C., Hall A., Whittaker D., Tuft S., Parry C. (2010). Minimum inhibitory concentrations of standard and novel antimicrobials for isolates from bacterial keratitis. Investig. Ophthalmol. Vis. Sci..

[55] Citron D.M., Tyrrell K.L., Merriam C.V., Goldstein E.J. (2012). In vitro activities of CB-183,315, vancomycin, and metronidazole against 556 strains of Clostridium difficile, 445 other intestinal anaerobes, and 56 Enterobacteriaceae species. Antimicrob. Agents Chemother..

[56] Grillon A., Schramm F., Kleinberg M., Jehl F. (2016). Comparative activity of ciprofloxacin, levofloxacin and moxifloxacin against *Klebsiella pneumoniae*, *Pseudomonas aeruginosa* and *Stenotrophomonas maltophilia* assessed by minimum inhibitory concentrations and time-kill studies. PLoS ONE.

[57] Kuti J.L., Wang Q., Chen H., Li H., Wang H., Nicolau D.P. (2018). Defining the potency of amikacin against *Escherichia coli*, *Klebsiella pneumoniae*, *Pseudomonas aeruginosa*, and *Acinetobacter baumannii* derived from Chinese hospitals using CLSI and inhalation-based breakpoints. Infect. Drug Resist..

[58] Michael C.A., Dominey-Howes D., Labbate M. (2014). The antimicrobial resistance crisis: Causes, consequences, and management. Front. Public Health.

[59] Frieri M., Kumar K., Boutin A. (2017). Antibiotic resistance. J. Infect. Public Health.

[60] Schechner V., Temkin E., Harbarth S., Carmeli Y., Schwaber M.J. (2013). Epidemiological interpretation of studies examining the effect of antibiotic usage on resistance. Clin. Microbiol. Rev..

[61] Zhu M., Jin Y., Duan Y., He M., Lin Z., Lin J. (2019). Multi-drug resistant Escherichia coli causing early-onset neonatal sepsis—A single center experience from China. Infect. Drug Resist..

[62] Klein E.Y., Van Boeckel T.P., Martinez E.M., Pant S., Gandra S., Levin S.A., Goossens H., Laxminarayan R. (2018). Global increase and geographic convergence in antibiotic consumption between 2000 and 2015. Proc. Natl. Acad. Sci. USA.

[63] Shyam R., Patel M.L., Kumar D. (2020). Etiology and outcome of patients with sepsis: A tertiary centre study. J. Med. Sci. Clin. Res..

[64] Chaurasia S., Sivanandan S., Agarwal R., Ellis S., Sharland M., Sankar M.J. (2019). Neonatal sepsis in South Asia: Huge burden and spiralling antimicrobial resistance. BMJ.

[65] Moran E., Munang M., Chan C., Chaudhri S., Himayakanthan M., Laird S., Moltu A., Naworynsky N., Pollard C., Saeed T. (2017). Sepsis quality standards are laudable but have low specificity. BMJ.

[66] Donnelly J.P., Safford M.M., Shapiro N.I., Baddley J.W., Wang H.E. (2017). Application of the Third International Consensus Definitions for Sepsis [Sepsis-3] Classification: A retrospective population-based cohort study. Lancet Infect. Dis..

[67] Oliver D. (2019). David Oliver: Sepsis—What’s behind the “hype”?. BMJ.

[68] Kuehn B.M. (2013). IDSA: Better, faster diagnostics for infectious diseases needed to curb overtreatment, antibiotic resistance. JAMA.

[69] Antibiotics. https://www.hopkinsmedicine.org/health/wellness-and-prevention/antibiotics.

[70] Sulis G., Adam P., Nafade V., Gore G., Daniels B., Daftary A., Das J., Gandra S., Pai M. (2020). Antibiotic prescription practices in primary care in low-and middle-income countries: A systematic review and meta-analysis. PLoS Med..

[71] Barker A.K., Brown K., Ahsan M., Sengupta S., Safdar N. (2017). Social determinants of antibiotic misuse: A qualitative study of community members in Haryana, India. BMC Public Health.

[72] Kotwani A., Joshi J., Lamkang A.S. (2021). Over-the-Counter Sale of Antibiotics in India: A Qualitative Study of Providers’ Perspectives across Two States. Antibiotics.

[73] Vercelli C., Gambino G., Amadori M., Re G. (2022). Implications of veterinary medicine in the comprehension and stewardsship of antimicrobial phenomenon: From the origins till nowadays. Vet. Anim. Sci..

[74] Yu L., Weize Y., Jie W. (2017). Guiding effect of serum procalcitonin [PCT] on the antibiotic application to patients with sepsis. Iran. J. Public Health.

[75] Schroeder S., Hochreiter M., Koehler T., Schweiger A.M., Bein B., Keck F.S., Von Spiegel T. (2009). Procalcitonin [PCT]-guided algorithm reduces length of antibiotic treatment in surgical intensive care patients with severe sepsis: Results of a prospective randomized study. Langenbeck’s Arch. Surg..

[76] Li Z., Yuan X., Yu L., Wang B., Gao F., Ma J. (2019). Procalcitonin-guided antibiotic therapy in acute exacerbation of chronic obstructive pulmonary disease: An updated meta-analysis. Medicine.

[77] Bellani G., Laffey J.G., Pham T., Fan E., Brochard L., Esteban A., Gattinoni L., Van Haren F., Larsson A., McAuley D.F. (2016). Epidemiology, patterns of care, and mortality for patients with acute respiratory distress syndrome in intensive care units in 50 countries. JAMA.

[78] Kwizera A., Baelani I., Mer M., Kissoon N., Schultz M.J., Patterson A.J., Musa N., Farmer J.C., Dünser M.W. (2018). The long sepsis journey in low- and middle-income countries begins with a first step... but on which road?. Crit. Care.

[79] Schultz M.J., Dünser M.W., Dondorp A.M., Adhikari N.K., Iyer S., Kwizera A., Lubell Y., Papali A., Pisani L., Riviello E.D. (2019). Current challenges in the management of sepsis in ICUs in resource-poor settings and suggestions for the future. Sepsis Management in Resource-Limited Settings.

[80] Phua J., Faruq M.O., Kulkarni A.P., Redjeki I.S., Detleuxay K., Mendsaikhan N., Sann K.K., Shrestha B.R., Hashmi M., Palo J.E.M. (2020). Critical care bed capacity in Asian countries and regions. Crit. Care Med..

[81] Booraphun S., Hantrakun V., Siriboon S., Boonsri C., Poomthong P., Singkaew B.O., Wasombat O., Chamnan P., Champunot R., Rudd K. (2021). Effectiveness of a sepsis programme in a resource-limited setting: A retrospective analysis of data of a prospective observational study [Ubon-sepsis]. BMJ Open.

[82] Shrestha G.S., Lamsal R., Tiwari P., Acharya S.P. (2021). Anesthesiology and Critical Care Response to COVID-19 in Resource-limited Settings: Experiences from Nepal. Anesthesiol. Clin..

[83] Popp W., Rasslan O., Unahalekhaka A., Brenner P., Fischnaller E., Fathy M., Goldman C., Gillespie E. (2010). What is the use? An international look at reuse of single-use medical devices. Int. J. Hyg. Environ. Health.

[84] Mer M., Schultz M.J., Adhikari N.K. (2017). Core elements of general supportive care for patients with sepsis and septic shock in resource-limited settings. Intensive Care Med..

[85] Duke T., Wandi F., Jonathan M., Matai S., Kaupa M., Saavu M., Subhi R., Peel D. (2008). Improved oxygen systems for childhood pneumonia: A multihospital effectiveness study in Papua New Guinea. Lancet.

[86] Barker A.K., Brown K., Siraj D., Ahsan M., Sengupta S., Safdar N. (2017). Barriers and facilitators to infection control at a hospital in northern India: A qualitative study. Antimicrob. Resist. Infect. Control.

[87] Permpikul C., Tongyoo S., Viarasilp T., Trainarongsakul T., Chakorn T., Udompanturak S. (2019). Early Use of Norepinephrine in Septic Shock Resuscitation (CENSER) A Randomized Trial. Am. J. Respir. Crit. Care Med..

[88] Bima P., Orlotti C., Smart O.G., Morello F., Trunfio M., Brazzi L., Montrucchio G. (2022). Norepinephrine may improve survival of septic shock patients in a low-resource setting: A proof-of-concept study on feasibility and efficacy outside the intensive care unit. Pathog. Glob. Health.

[89] Li Q., Guan X., Wu P., Wang X., Zhou L., Tong Y., Ren R., Leung K.S., Lau E.H., Wong J.Y. (2020). Early transmission dynamics in Wuhan, China, of novel coronavirus—Infected pneumonia. N. Engl. J. Med..

[90] Jain V.K., Iyengar K.P., Vaishya R. (2021). Differences between First wave and Second wave of COVID-19 in India. Diabetes Metab. Syndr..

[91] https://covid19.who.int/region/searo/country/lk.

[92] Huang C., Wang Y., Li X., Ren L., Zhao J., Hu Y., Zhang L., Fan G., Xu J., Gu X. (2020). Clinical features of patients infected with 2019 novel coronavirus in Wuhan, China. Lancet.

[93] Damiani M., Gandini L., Landi F., Borleri G., Fabretti F., Gritti G., Riva I. (2021). Extracorporeal cytokine hemadsorption in severe COVID-19 respiratory failure. Respir. Med..

[94] Mehta P., McAuley D., Brown M., Sanchez E., Tattersall R., Manson J. (2020). COVID-19: Consider cytokine storm syndromes and immunosuppression. Lancet.

[95] Tang Y., Liu J., Zhang D., Xu Z., Ji J., Wen C. (2020). Cytokine storm in COVID-19: The current evidence and treatment strategies. Front. Immunol..

[96] Zhang B., Zhou X., Qiu Y., Song Y., Feng F., Feng J., Song Q., Jia Q., Wang J. (2020). Clinical characteristics of 82 cases of death from COVID-19. PLoS ONE.

[97] (2021). As Covid-19 Devastates India, Deaths Go Undercounted. N. Y. Times.

[98] Ghonimi T.A.L., Alkad M.M., Abuhelaiqa E.A., Othman M.M., Elgaali M.A., Ibrahim R.A.M., Joseph S.M., Al-Malki H.A., Hamad A.I. (2021). Mortality and associated risk factors of COVID-19 infection in dialysis patients in Qatar: A nationwide cohort study. PLoS ONE.

[99] Yam E.L.Y., Hsu L.Y., Yap E.P.H., Yeo T.W., Lee V., Schlundt J., Lwin M.O., Limmathurotsakul D., Jit M., Dedon P. (2019). Antimicrobial Resistance in the Asia Pacific region: A meeting report. Antimicrob. Resist. Infect. Control.

[100] Ray S., Goyal S. (2020). Precision medicine: From concept to clinical practice—A promising challenge!. J. Mar. Med. Soc..

[101] da Silva F.P., Machado M.C.C. (2015). Personalized medicine for sepsis. Am. J. Med. Sci..

[102] Chong H.Y., Allotey P.A., Chaiyakunapruk N. (2018). Current landscape of personalized medicine adoption and implementation in Southeast Asia. BMC Med. Genom..

[103] Evangelatos N., Bauer P., Reumann M., Satyamoorthy K., Lehrach H., Brand A. (2017). Metabolomics in sepsis and its impact on public health. Public Health Genom..

[104] Podder V., Dhakal B., Shaik G.U.S., Sundar K., Sivapuram M.S., Chattu V.K., Biswas R. (2018). Developing a case-based blended learning ecosystem to optimize precision medicine: Reducing overdiagnosis and overtreatment. Healthcare.

[105] https://www.ahrq.gov/sites/default/files/wysiwyg/antibiotic-use/best-practices/sepsis-facilitator-guide.pdf.

[106] Dunser M.W., Festic E., Dondorp A., Kissoon N., Ganbat T., Kwizera A., Haniffa R., Baker T., Shultz M.J. (2012). Recommendations for sepsis management in resource-limited settings. Intensive Care Med..

[107] Ali J., Vuylsteke A. (2019). Extracorporeal membrane oxygenation: Indications, technique and contemporary outcomes. Heart.

[108] Gotur D.B. (2017). Sepsis Diagnosis and Management. J. Med. Sci. Health.

[109] Bonavia A., Groff A., Karamchandani K., Singbartl K. (2018). Clinical utility of extracorporeal cytokine hemoadsorption therapy: A literature review. Blood Purif..

[110] Cytosorbent Corporation, CytoSorb Fields of Application. https://cytosorb-therapy.com/en/the-therapy/fields-of-application/.

[111] https://cytosorb-therapy.com/en/covid-19/.

[112] CytoSorbents Corporation, CytoSorb: Broad Cytokine and Toxin Reduction to Control Deadly Inflammation. http://cytosorbents.com/products/cyto-sorb/.

[113] Schultz P., Schwier E., Eickmeyer C., Henzletr D., Kohler T. (2021). High dose CytoSorb haemadsorption is associated with improved survival in patients with septic shock: A retrospective cohort study. J. Crit. Care.

[114] https://cytosorb-therapy.com/en/the-therapy/.

[115] Basu R., Pathak S., Goyal J., Chaudhry R., Goel R.B., Barwal A. (2014). Use of a novel hemoadsorption device for cytokine removal as adjuvant therapy in a patient with septic shock with multi-organ dysfunction: A case study. Indian J. Crit. Care Med. Peer-Rev. Off. Publ. Indian Soc. Crit. Care Med..

[116] Paul R., Sathe P., Kumar S., Prasad S., Aleem M., Sakhalvalkar P. (2021). Multicentered prospective investigator initiated study to evaluate the clinical outcomes with extracorporeal cytokine adsorption device [CytoSorb^®^] in patients with sepsis and septic shock. World J. Crit. Care Med..

[117] Cytosorbents Corporation (2018). CytoSorb Literature Database.

[118] Rizvi S., Danic M., Silver M., LaBond V. (2021). Cytosorb filter: An adjunct for survival in the COVID-19 patient in cytokine storm? A case report. Heart Lung.

[119] Alharthy A., Faqihi F., Memish Z.A., Balhamar A., Nasim N., Shahzad A., Tamim H., Alqahtani S.A., Brindley PGKarakitsos D. (2021). Continuous renal replacement therapy with the addition of CytoSorb cartridge in critically ill patients with COVID-19 plus acute kidney injury: A case-series. Artif. Organs.

[120] Mehta Y., Mehta C., Nanda S., Kochar G., George J.V., Singh M.K. (2021). Use of CytoSorb therapy to treat critically ill coronavirus disease 2019 patients: A case series. J. Med. Case Rep..

[121] Song T., Hayanga J., Durham L., Garrison L., McCarthy P., Barksdale A., Smith D., Bartlett R., Jaros M., Nelson P. (2021). CytoSorb therapy in COVID-19 (CTC) patients requiring extracorporeal membrane oxygenation: A multicentric retrospective registry. Front. Med..

[122] Kumar S., Damera S. (2020). Paediatric patient with dengue fever and associated multi-organ dysfunction syndrome (MODS) receiving hemoadsorption using Cytosorb^®^ A case report on clinical experience. IJMDAT.

[123] Khan Z.A. (2016). A Clinical Experience Of Using Extracorporeal Cytokine Adsorption Device [Cytosorb^®^] In A Case Of Dengue Fever. J. Evid. Based Med. Healthc..

[124] Krishan K., Dutta R., Chand R., Malhotra R. (2020). Experience of using an extracorporeal cytokine hemoadsorber [CytoSorb^®^] in systemic inflammatory response syndrome after heart transplantation. Indian J. Transplant..

[125] Mehta Y., Singh A., Singh A., Gupta A., Bhan A. (2021). Modulating the Inflammatory Response with Hemadsorption [CytoSorb] in Patients Undergoing Major Aortic Surgery. J. Cardiothorac. Vasc. Anesth..

[126] Padiyar S., Deokar A., Birajdar S., Walawalkar A., Doshi H. (2019). Cytosorb for management of acute kidney injury due to rhabdomyolysis in a child. Indian Paediatr..

[127] Sairam R. Safety and Efficacy of Cytosorb Hemadsorption in Children with Multiorgan Dysfunction Syndrome. https://www.ijccm.org/doi/IJCCM/pdf/10.5005/ijccm-24-S2-S1.

[128] https://literature.cytosorb-therapy.com/infoitem/use-of-cytosorb-in-a-patient-diagnosed-with-sepsis-and-mods-due-to-infection-with-salmonella-typhi?lang=.

[129] http://cytosorb-therapy.com/wp-content/uploads/2016/01/CaseStudy_Booklet_10-001-20_EN_241115_low.pdf.

[130] https://literature.cytosorb-therapy.com/infoitem/combined-application-of-cytosorb-and-sustained-low-efficiency-dialysis-sled-in-a-patient-with-septic-shock-and-multiple-organ-failure?lang=en.

[131] Mehta Y., Mehta C., Kumar A., George J.V., Gupta A., Nanda S., Kochhar G., Raizada A. (2020). Experience with hemoadsorption [CytoSorb^®^] in the management of septic shock patients. World J. Crit. Care Med..

[132] Singh Y.P., Chhabra S.C., Lashkari K., Taneja A., Garg A., Chandra A., Kochhar G., Jain S. (2020). Hemoadsorption by extracorporeal cytokine adsorption therapy [CytoSorb^®^] in the management of septic shock: A retrospective observational study. Int. J. Artif. Organs.

[133] Brouwer W.P., Duran S., Kuijper M., Ince C. (2019). Hemadsorption with CytoSorb shows a decreased observed versus expected 28-day all-cause mortality in ICU patients with septic shock: A propensity-score-weighted-retrospective study. Crit. Care.

[134] Rugg C., Klose R., Hornung R., Innerhofer N., Bachler M., Schmid S., Fries D., Strohle M. (2020). Hemadsorption with CytoSorb in septic shock reduces catecholamine requirements and in-hospital mortality: A single centre retrospective genetic matched analysis. Biomedicines.

[135] Kogelmann K., Hubner T., Schwameis F., Druner M., Scheller M., Jarczak D. (2021). First evaluation of new dynamic scoring system intended to support prescription of adjuvant CytoSorb hemadsorption therapy in patients with septic shock. J. Clin. Med..

[136] Nassiri A.A., Hakemi M.S., Shahrami R., Koomleh A.A., Sabaghian T. (2021). Blood purification CytoSorb in critically-ill COVID-19 patients: A case series of 26 patients. Artif. Organs.

[137] Wunderlich-Sperl F., Kautzky S., Pickem C., Hormann C. (2021). Adjuvant hemadsorption therapy in patients with severe COVID-19 and related organ failure requiring CRRT or ECMO therapy: A case series. Int. J. Artif. Organs.

[138] Hawcher F., Laszlo I., Oveges N., Trasy D., Ondrik Z., Molnar Z. (2019). Extracorporeal cytokine adsorption in septic shock: A proof of concept randomized, controlled pilot study. J. Crit. Care.

[139] Schadler D., Pausch C., Heise D., Meier-Hellmann A., Brederlau J., Weiler N., Marx G., Putensen C., Spies C., Jorres A. (2017). The effect of a novel extracorporeal cytokine hemadsorption device on IL-6 elimination in septic patients: A randomized controlled trial. PLoS ONE.

[140] Vincent J.-L., Singer M., Einav S., Moreno R., Wendon J., Teboul J.-L., Bakker J., Hernandez G., Annane D., de Man A.M. (2021). Equilibrating SSC guidelines with individualized care. Crit. Care.

